# A New 3-Ketosteroid-Δ^1^–Dehydrogenase with High Activity and Broad Substrate Scope for Efficient Transformation of Hydrocortisone at High Substrate Concentration

**DOI:** 10.3390/microorganisms10030508

**Published:** 2022-02-25

**Authors:** Yu Wang, Rui Zhang, Jinhui Feng, Qiaqing Wu, Dunming Zhu, Yanhe Ma

**Affiliations:** National Technology Innovation Center of Synthetic Biology, National Engineering Laboratory for Industrial Enzymes, Tianjin Engineering Research Center of Biocatalytic Technology, Tianjin Institute of Industrial Biotechnology, Chinese Academy of Sciences, Tianjin Airport Economic Area, 32 Xi Qi Dao, Tianjin 300308, China; wang_y1@tib.cas.cn (Y.W.); zhang_r@tib.cas.cn (R.Z.); feng_jh@tib.cas.cn (J.F.); ma_yh@tib.cas.cn (Y.M.)

**Keywords:** 3-ketosteroid-Δ^1^–dehydrogenase, Δ^1^-dehydrogenation, steroid biotransformation, hydrocortisone

## Abstract

3-Ketosteroid-Δ^1^-dehydrogenases (KstDs [EC 1.3.99.4]) catalyze the Δ^1^-dehydrogenation of steroids and are a class of important enzymes for steroid biotransformations. In this study, nine putative *kstD* genes from different origins were selected and overexpressed in *Escherichia coli* BL21(DE3). These recombinant enzymes catalyzed the Δ^1^-desaturation of a variety of steroidal compounds. Among them, the KstD from *Propionibacterium* sp. (PrKstD) displayed the highest specific activity and broad substrate spectrum. The detailed catalytic characterization of PrKstD showed that it can convert a wide range of 3-ketosteroid compounds with diverse substituents, ranging from substituents at the C9, C10, C11 and C17 position through substrates without C4-C5 double bond, to previously inactive C6-substituted ones such as 11β,17-dihydroxy-6α-methyl-pregn-4-ene-3,20-dione. Reaction conditions were optimized for the biotransformation of hydrocortisone in terms of pH, temperature, co-solvent and electron acceptor. By using 50 g/L wet resting *E. coli* cells harboring PrKstD enzyme, the conversion of hydrocortisone was about 92.5% within 6 h at the substrate concentration of 80 g/L, much higher than the previously reported results, demonstrating the application potential of this new KstD.

## 1. Introduction

Steroids have important physiological functions with a wide range of clinical applications, and they are currently the second largest class of drugs after antibiotics [1]. Among the steroidal drugs, many contain a double bond at the 1,2-position because they usually have enhanced potency and cause less drug-induced salt retention. For example, the anti-inflammatory power of prednisolone is four times stronger than that of hydrocortisone, while that of prednisone is five times that of cortisone [2]. Budesonide, an asthma medication of the corticosteroid type with C_1_=C_2_ double bond, had a market value of $1.01 billion as Pulmicort and $2.72 billion as Symbicort (co-marketed with Formoterol) in 2020 (https://njardarson.lab.arizona.edu/content/top-pharmaceuticals-poster, accessed date, 5 December 2021). Accordingly, Δ^1^-dehydrogenation of steroid compounds is an important reaction in the synthesis of medically useful steroidal molecules [3,4,5,6]. Such a transformation may be achieved by chemical or biocatalytic methods. While the chemical methods require a complicated series of reaction steps due to the complex nature of the steroidal molecules [7,8,9], biocatalytic Δ^1^-dehydrogenation of steroids is usually performed under mild conditions with high regioselectivity [5,6,10].

3-Ketosteroid-Δ^1^-dehydrogenase (KstD) catalyzes Δ^1^-dehydrogenation and has been well studied for its prominent role in steroid catabolism [11,12,13,14]. On the one hand, KstD inactivation was achieved in many actinomycetes species including *Mycobacterium* and *Rhodococcus* species, leading to the production of important intermediates such as 4-androstene-3,17-dione (AD) and 9α-hydroxy-4-androsten-3,17-dione (9α-OH-AD)[15,16,17] by preventing the degradation of the steroid nuclei. On the other hand, genetic manipulations were performed with *Mycobacterium* and *Arthrobacter* species to enhance the expression of KstD-encoding genes, yielding the recombinant strains with improved Δ^1^-dehydrogenation efficiency [18,19,20,21].

Superior 3-ketosteroid-1-dehydrogenases were identified from *Nocardioides simplex* VKM Ac-2033D, and the relative strains are well-established microbial catalysts for steroid 1-dehydrogenation [22]. Δ^1^-KSTDs from several microorganisms were overexpressed in *Bacillus* species [23,24,25], *Corynebacterium* *crenatum* [26], *Escherichia coli* [21,27,28,29,30,31,32], or *Pichia pastoris* [27] to enable Δ^1^-dehydrogenation of various valuable steroid intermediates. This approach has drawn increasing attention because it avoids the by-product accumulation in the microbial transformation using the wild-type microorganisms as the biocatalyst due to other active enzymes in the microorganisms. However, given the high demand for effective KstD-catalyzed Δ^1^-dehydrogenation by the pharmaceutical steroid industry, new enzymes with high activity and wide substrate spectrum for effectively converting valuable steroid intermediates are greatly appreciated. In this work, a new KstD enzyme from *Propionibacterium* sp (PrKstD) was purified and characterized, and its substrate scope and application in the synthesis of prednisolone were explored.

## 2. Materials and Methods

### 2.1. Reagents, Strains, and Media

All reagents were at least of analytical grade unless otherwise noted. 2,6-Dichloro-phenolindophenol (DCPIP) and phenazine methosulfate (PMS) were supplied by Shanghai Yuanye Bio-Technology Co., Ltd. (Shanghai, China). 17β-Hydroxy-5α-androstan-3-one and testosterone was obtained from Shanghai Meryer Chemical Technology Co., Ltd. (Shanghai, China). Cortexolone and FAD were purchased from Tokyo Chemical Industry Co., Ltd. Other steroids were donated by Zhejiang Xianju Junye Pharmaceutical Co., Ltd. (Zhejiang, China) and Tianjin Tianyao Pharmaceuticals Co., Ltd. (Tianjin, China). All other chemicals were from commercial sources. Restriction endo-nucleases, T4 DNA ligases, and protein marker were obtained from Thermo Fisher Scientific (Waltham, MA, USA). FastPfu DNA polymerase was purchased from TransGen Biotech Co., Ltd. (Beijing, China).

### 2.2. Gene Cloning and Expression

Nine putative KstDs from different origins were selected in this study (Table 1). The gene sequences of PrKstD, JkKstD, StpKstD, AcKstD and PseKstD were optimized through codon optimization at http://www.prodoric.de/JCat (accessed date, 10 September 2020), and synthesized and cloned into NdeI and HindIII sites of plasmid pET21a(+). The gene sequences of SatKstD and NvKstD were cloned from genomic DNA of *Saccharopolyspora kobensis* and *Nocardia nova* with the primers shown in Table S1. The phylogenetic tree was constructed using the MEGA version 4.0 software package with ClustalW and the neighbor-joining algorithm [20]. Multiple sequence alignments were performed to find similarities and differences using DNAMAN.

*Saccharopolyspora kobensis* and *Nocardia nova* were cultivated in Luria-Bertani (LB) broth at 30 °C with shaking (200 rpm). Genomic DNAs were prepared by using the TianGen Bacteria DNA Kit DP302 (TianGen Biotech, Beijing, China) according to manufacturer’s instructions. The DNA fragment of *Sat-kstD* gene was obtained by PCR using FastPfu DNA polymerase. The following PCR program was used: 98 °C for 2 min followed by 30 cycles of 30 s at 98 °C, 30 s at 60 °C, 2 min at 72 °C, and then 72 °C for 5 min. The PCR product of *Sat-kstD* was cloned into NdeI/HindIII-digested pET21a(+). The plasmid, pET21a(+)-Sat-kstD, was introduced into *E. coli* BL21 (DE3), resulting in recombinant strain pET21a(+)-Sat-kstD-BL21. Similarly, other *kstD* genes were also cloned in pET21a(+) vector and transformed into *E. coli* BL21 (DE3).

The recombinant strains were cultured at 37 °C in LB medium (pH 7.0) containing ampicillin (100 mg/L) or kanamycin (50 mg/L) until the optical density (OD) reached 0.6~1.0 at 600 nm. Subsequently, induction by the addition of 0.1 mM IPTG was performed for 16 h at 25 °C and the cells were harvested by centrifugation.

### 2.3. Enzyme Activity Assay of Crude Enzymes

**Figure 1:**
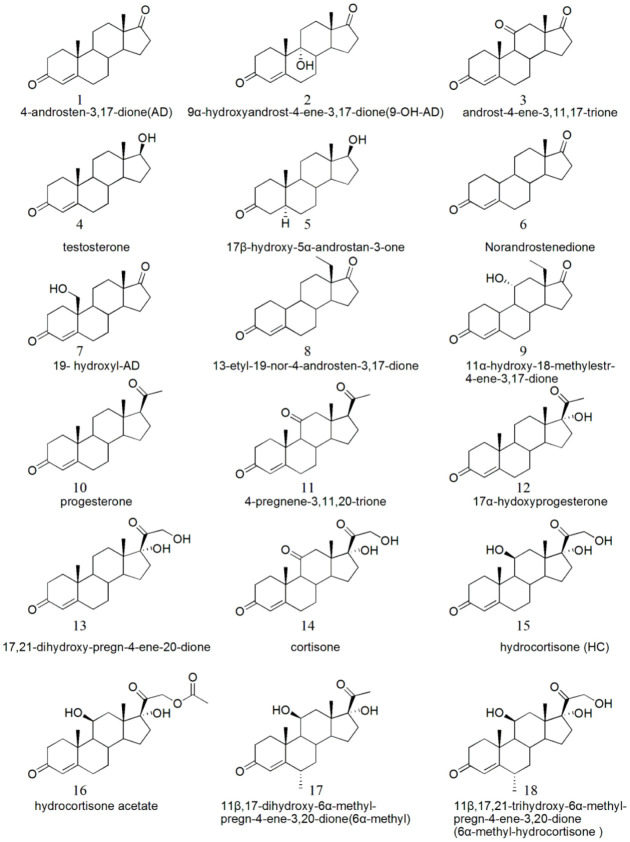
Structures of the steroids tested in this study.

The recombinant cells were resuspended in 50 mM Tris-HCl buffer (pH 8.0) and disrupted by 260 W ultrasonic power for 3 min. Cell debris was precipitated by centrifugation at 10,000× *g* for 20 min. The supernatants (cell-free extracts) were used for activity assays with a range of substrates as described below (Figure 1).

The enzyme activity was measured spectrophotometrically at 30 °C. The reaction mixture (200 μL) consisted of 50 mM Tris−HCl (pH 8.0), 1.5 mM phenazine methosulfate (PMS), 150 μM 2,6-dichloro-phenolindophenol (DCPIP), an appropriate amount of crude cell-free extracts, and 0.4–1.25 mM substrates in methanol (2–5%, v/v). The decrease in absorbance at 600 nm (ε _600 nm_ = 18.7 × 10^3/^(cm·M) was monitored using the SpectraMax M2 microplate reader (Molecular Devices) [20]. Protein concentrations were determined using the BCA Protein Assay Kit. One unit of enzyme activity (U) is defined as the reduction of 1 μmol of DCPIP per minute at 30 °C, pH 8.0. Specific activities are defined as micromoles per milligram per minute (U/mg). The experiments were carried out in triplicate.

### 2.4. Purification and Characterization of PrKstD

The His-tagged recombinant proteins were purified by Ni^2+^−chelating Sepharose affinity chromatography. All steps were carried out at 4 °C unless otherwise indicated. The cell pellet was suspended in lysis buffer (20 mM Tris-HCl, pH 8.0, 500 mM NaCl and 1 mM dithiothreitol (DTT)). The cell suspension was lysed by high-pressure homogenizer (APV 1000) and centrifuged at 10,000× *g* for 30 min. The supernatant was applied to the Ni^2+^−column followed by washing with buffer A (20 mM Tris-HCl, pH 8.0, 500 mM NaCl, 30 mM imidazole, 20 µM FAD and 1 mM DTT). Elution was carried out using a linear imidazole gradient (30–500 mM) in buffer A. The purified PrKstD was identified on SDS-PAGE with Image Lab ™ Software version 5.2.1 (Bio-Rad, Hercules, CA, USA) (Figure S1). The yellow-colored PrKstD was exchanged into buffer (20 mM Tris–HCl (pH 8.0), 200 mM NaCl and 20 µM FAD) via ultrafiltration (30 kDa molecular weight cut-off), finally diluted with the same buffer to 3.2 mg L^−1^ for the enzyme activity assay.

Hydrocortisone (0.5 mM) was used as the substrate for the characterization of PrKstD. The optimal pH for purified PrKstD was determined at 30 °C using different reaction buffers, which contained 50 mM potassium phosphate, Tris–HCl or Gly–NaOH in the pH range of 6.0–10.0. pH stability was determined by incubating the enzyme at 4 °C for 2 h and the residual activity was measured at pH 8.0 and 30 °C. PrKstD activity at 30 °C, potassium phosphate buffer pH 8.0 was defined as 100%.

To determine the optimal temperature, the activity assay was performed at various temperatures from 25 to 45 °C in 50 mM potassium phosphate buffer (pH 8.0). PrKstD activity at 30 °C, potassium phosphate buffer pH 8.0 was defined as 100%. The thermostability was determined at 30 °C by measuring the residual activity, after the incubation of the enzyme in potassium phosphate buffer (50 mM, pH 8.0) at different temperatures (30–50 °C) for 1, 2, 3 and 4 h, and then cooling the mixture on ice for 10 min. The PrKstD activity before incubation was defined as 100%.

The kinetic parameters of the KstD enzyme were determined by incubating the respective purified enzyme and varying concentrations of steroid substrate as described above. *K*_m_,V_max_ values were obtained by fitting the Michaelis–Menten equation to the data and *K*i value was obtained by fitting the Substrate Inhibition (Uncompetitive) equation to the data using the SigmaPlot program (version 12.0). The experiments were carried out in triplicate.

### 2.5. Optimization of Process Conditions for the Bioconversion of Hydrocortisone to Prednisolone

The recombinant *E. coli* strain expressing PrKstD was cultured at 37 °C in LB medium (pH 7.0) containing ampicillin (100 mg/L) until the OD_600_ reached 0.6~0.8. Subsequently, induction with 0.1 mM IPTG was performed for 16 h at 25 °C. Cells were harvested by centrifuge (4000× *g*).

For determining optimal pH, cells were resuspended in different reaction buffers, which contained 50 mM potassium phosphate, Tris–HCl or Gly–NaOH in the pH range of 6.0–10.0. The biotransformation was performed in 4 mL reaction volume in a little glass bottle (15 mL) containing 1 mM PMS and 40 g/L hydrocortisone and 50 g/L wet cells at 30 °C with shaking at 200 rpm. The reaction mixture was extracted with ethyl acetate and evaporated to dryness. The residual was dissolved in methanol and was subjected to high-performance liquid chromatography (HPLC) analysis to determine the conversion of hydrocortisone. HPLC analyses were carried out on an Agilent 1200 system and an Agilent Eclipse XDB–C18 column (5 μm particles, 250 mm × 4.6 mm) was used with a mobile phase consisting of 30% acetonitrile and 70% water (v/v) at a flow rate of 0.6 mL/min. The UV absorbance was determined at 254 nm, and the column temperature was at 30 °C. The retention times were compared to those of authentic hydrocortisone and prednisolone samples. ^1^H-NMR analysis indicated that the product was identical with authentic prednisolone sample.

Different co-solvents were used in this study such as dimethyl sulfoxide (DMSO), 1,4-dioxane, dimethyl formamide (DMF), methanol, ethanol, isopropyl alcohol, tetrahydrofuran (THF) as shown in Table S2, Tween 80, β-cyclodextrin (β-CD), randomly methylated-β-cyclodextrin (RM-β-CD) and hydroxypropyl-β-cyclodextrin (HP-β-CD). The reaction mixture containing 50 g/L wet cells as described above, 40 g/L hydrocortisone (suspended in 5% organic solvent (v/v) or 0.1% Tween 80 (w/v) or CDs with molar ratio to hydrocortisone 1:1) and 1 mM PMS in 4 mL of Tris-HCl buffer (pH 8.0, 50 mM) in a 15 mL glass bottle was incubated at 30 °C with shaking at 200 rpm. Besides, the effect of different methanol concentration on the hydrocortisone biotransformation was also investigated. Similarly, the reaction was performed with different final concentrations of PMS (0, 0.1, 0.2, 0.5, 1, 3, 5, 8, 10 mM) in Tris-HCl buffer (pH 8.0, 50 mM). The reaction was shaken at 30 °C with shaking at 200 rpm, worked-up and analyzed as described above.

Under the optimized process conditions, the time course of bioconversion of hydrocortisone by recombinant whole cells was determined in a 100 mL flask. The mixture containing 3 mM PMS, 50 g/L wet cells and different hydrocortisone concentrations (40, 50, 60, 70, 80 g/L, 5% methanol, v/v) in Tris-HCl buffer (pH 8.0, 50 mM) with the final volume about 20 mL was incubated at 30 °C with shaking at 200 rpm. As the hydrocortisone was not completely soluble in aqueous solution of methanol (5%, v/v), at the beginning of the reaction, the substrate was suspended on the surface, and as the reaction proceeded, the substrate would disperse in the reaction medium. Samples were withdrawn at different time intervals, worked-up and analyzed as described above. The experiments were carried out in triplicate.

## 3. Results

### 3.1. Δ1-Dehydrogenation of Steroids by Cell-Free Extracts of Various KstD Enzymes

In order to search for KstDs with high activity and wide substrate spectrum, nine KstDs from different origins (Table 1) were selected and their genes were cloned or synthesized and overexpressed in *E. coli* BL21(DE3). These KstD enzymes were screened with representative steroids in Figure 1 with diverse substituents, ranging from substituents at the C6, C9, C11 and C17 position to substrates without C4–C5 double bond, in an attempt to find enzymes with high activity and wide substrate spectrum. As shown in Table 2, all of these enzymes showed Δ^1^-dehydrogenation ability. Among them, PrKstD displayed the highest specific activity and wide substrate profile, which was active toward all tested substrates.

As shown in Table 2, introducing a hydroxyl group at the C9 position reduced the enzyme activity for most of the enzymes, but not for JkKstD. The presence of a functional group (hydroxyl or carbonyl group) at C11 had a great negative effect on the dehydrogenase activity of PseKstD, JkKstD, NvKstD, NnKstD and MsKstD4, especially for the hydroxyl group. However, for PrKstD, SatKstD and AcKstD, substituent at the C11 position of the substrates exerted little influence on the dehydrogenase activity. Most of the enzymes showed lower activity toward progesterone (10) than AD (1), suggesting that an acetyl group at the C-17 position of the steroidal substrates reduced the enzyme activity. When the steroid A-ring is saturated, e.g., 5α–T (5), the dehydrogenase activity was greatly decreased except PseKstD and MsKstD4. The majority of enzymes, except PrKstD, SatKstD and StpKstD, were inactive toward steroidal substrates having a methyl substituent at the C6 position such as 6α-methyl (17), which may be due to the steric effect of the methyl group. As a whole, the KstD activity was significantly dependent on the individual enzyme and the substrate structures.

### 3.2. Biochemical Properties and Kinetic Parameters of Purified PrKstD

**Figure 2:**
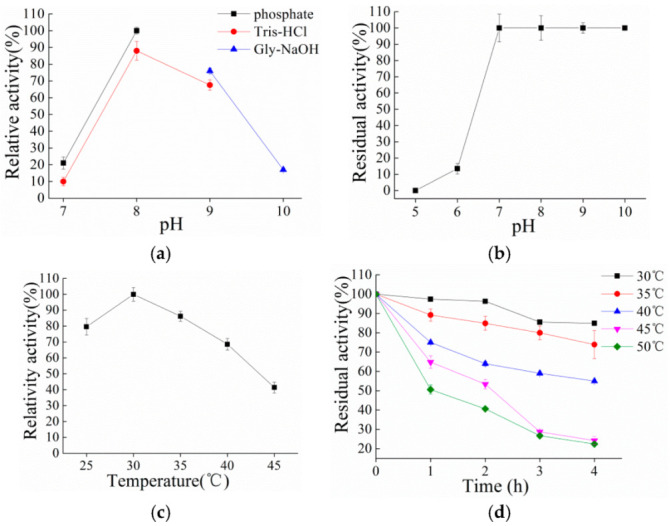
Effect of the pH and temperature on the activity and stability of recombinant PrKstD. (**a**,**b**) Effects of pH on recombinant PrKstD activity and stability. Assays were performed at 30 °C using different reaction buffers. (**c**,**d**) Effect of temperature on recombinant PrKstD activity and stability. Assays were performed at various temperatures in 50 mM potassium phosphate buffer (pH 8.0).

To further study the catalytic properties of PrKstD, its purification was performed by Ni^2+^ column chromatography. The protein was purified and the molecular mass of 55 kDa was confirmed by SDS-PAGE (Table S3, Figure S1).The purified PrKstD was active at pH 7.0–10.0 and the maximal activity was observed in phosphate buffer at pH 8.0. The PrKstD exhibited good pH stability over the pH range of 7.0–10.0 and nearly lost the activity at pH 5.0 and 6.0 (Figure 2a,b). The optimal temperature for PrKstD activity was 30 °C. The activity began to decrease dramatically when the temperature was over 40 °C. PrKstD exhibited good thermal stability at 30–35 °C and still retained more than 80% activity after 3 h. There was 20% residual activity after pre-incubation at 50 °C for 4 h (Figure 2c,d).

To investigate the substrate specificity of PrKstD, the substrate library was further expanded, as shown in Figure 1, and the kinetic parameters of purified PrKstD toward these steroids were measured. The results are summarized in Table 3.

PrKstD displayed the highest catalytic efficiency (*k*_cat_/*K*_m_) toward AD (1, 24.9 × 10^6^ M^−1^s^−1^ ). Higher *K*_m_ (21.5 μm) and lower *k*_cat_ (147.6 s^−^^1^) than that of AD were obtained for 9α-OH-AD (2) as a substrate, indicating that the hydroxyl group at C9 reduced the activity of PrKstD. A saturated A-ring, e.g., 5α–T (5), exerted negative influence both on substrate affinity (36.6 μM) and reaction rate (61.1 s^−^^1^). The catalytic efficiency of PrKstD decreased significantly for the substrates norandrostenedione (6) and 19-hydroxyl-AD (7) due to the importance of the 10β-methyl group, which is possibly recognized by the enzyme, since in the crystal structure of Δ^1^-KSTD1•ADD [33], the 10β-methyl group is at van der Waals distance to the Phe-116 and Phe-294 side chains.

The *k*_cat_/*K*_m_ for progesterone was 4.3 times lower than on AD, which implied that PrKstD reacted better on steroidal substrates with a C17 carbonyl group (e.g., AD; 1) than an acyl substituent (e.g., progesterone; 10). When the C11 position was substituted by the hydroxy group, substrate affinity of PrKstD for hydrocortisone (15, *K*_m_ = 164.5 μM) was 7.9 times lower than that of cortexolone (13, *K*_m_ = 20.8 μM). The similar catalytic efficiency was observed for hydrocortisone acetate (16) and hydrocortisone (15), indicating the acetyl group at C21 had no effect on the dehydrogenase activity of PrKstD. A methyl substituent at the C6 position greatly influenced the catalytic activity of KstD enzymes, since MsKsdD1 from *M. smegmatis* mc^2^ 155 and ReKstD from *Rhodococcus erythropolis* were inactive on 6α-methyl (17) [29]. PrKstD was active on 6α-methyl (17) and 6α-methyl-hydrocortisone (18), but with much lower catalytic efficiency.

### 3.3. Optimization of Process Conditions for the Bioconversion of Hydrocortisone to Prednisolone

**Figure 3:**
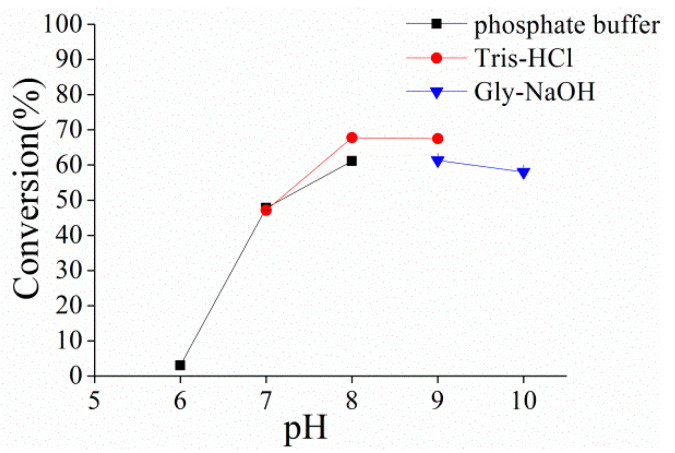
Effect of pH on the Δ^1^-dehydrogenation of hydrocortisone to prednisolonecatalyzed by resting cells containing PrKstD. The biotransformation was performed in different reaction buffers containing 1 mM PMS and 40 g/L hydrocortisone and 50 g/L wet cells at 30 °C.

The conversion of hydrocortisone to prednisolone is an important reaction due to its commercial interest [34]. The conversion rates using 40 g/L of hydrocortisone and 50 g/L of wet cells were measured at different pH. The conversion was <10% when the pH was below 6.0 and improved with the increase of buffer pH from 6.0 to 8.0. The highest conversion was 70% after 1.5 h and was obtained at pH 8.0 in Tris-HCl (Figure 3).

**Figure 4:**
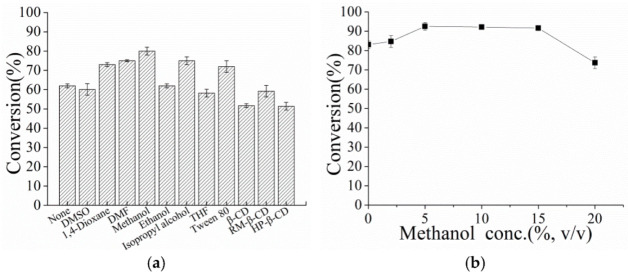
The Effects of Co-solvent on the Δ^1^-dehydrogenation of hydrocortisone to prednisolone catalyzed by resting cells containing PrKstD. (**a**) Effects of different co-solvents. (**b**) Effect of various methanol concentrations. The biotransformation was performed in Tris-HCl buffer (pH 8.0, 50 mM) containing 50 g/L wet cells, 40 g/L hydrocortisone and 1 mM PMS at 30 °C.

The observed reaction rate and an overall reaction yield strongly depend on the availability of hydrophobic steroids in the reaction medium [35]. The bioconversion of hydrocortisone to prednisolone by the resting cells was carried out in the presence of various co-solvents (Figure 4a). The highest conversion (80%) of hydrocortisone was obtained when methanol served as the co-solvent, followed by DMF and isopropanol with about 75% conversion. The effect of different concentrations of methanol on the bioconversion of hydrocortisone was also studied (Figure 4b). The highest conversion was achieved with 5–15% (v/v) methanol at the concentration of 40 g/L hydrocortisone. A further increase in methanol concentration did not benefit the transformation.

**Figure 5:**
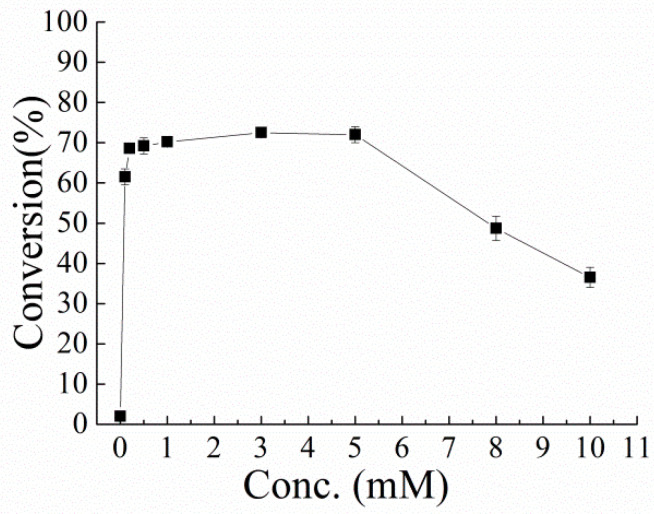
Transformation of hydrocortisone by resting cells containing PrKstD with different concentrations of phenazine methosulfate (PMS). The reaction was performed with different final concentrations of PMS (0, 0.1, 0.2, 0.5, 1, 3, 5, 8, 10 mM) in Tris-HCl buffer (pH 8.0, 50 mM) at 30 °C.

The oxidation of ketosteroids by KstDs results in a reduction of the FAD cofactor. To close the catalytic cycle, the reduced form of FAD has to be reoxidized by an external oxidant. In most studies, phenazine methosulfate (PMS) are used for such purposes [29,30,36,37]. As shown in Figure 5, the Δ^1^-dehydrogenation of hydrocortisone could not proceed without PMS. Since the electron acceptor PMS could be regenerated by molecular oxygen [38], a catalytic concentration (5 mM) was enough for enzyme regeneration. Higher PMS concentration had a negative effect on the transformation. This may be due to the enzyme inhibition by PMS with an inhibitor constant *K*i of 2.2 mM, which was measured and is shown in Table S4.

**Figure 6:**
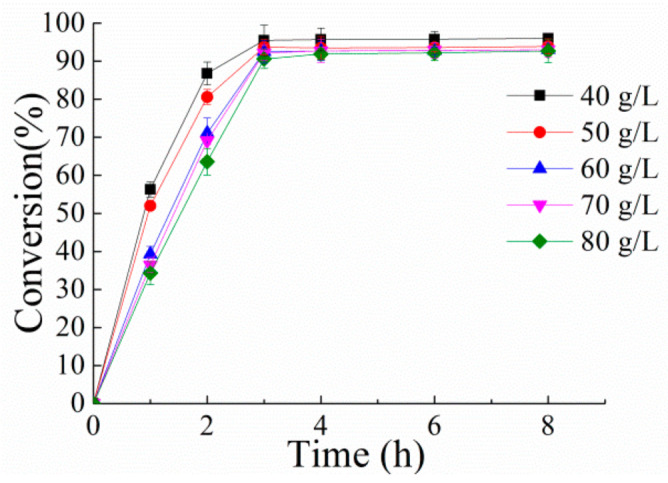
Time course of bioconversion of different hydrocortisone concentrations by resting cells containing PrKstD. The reaction mixture containing 3 mM PMS, 50 g/L wet cells and different hydrocortisone concentrations (40, 50, 60, 70, 80 g/L, 5% methanol, v/v) in Tris-HCl buffer (pH 8.0, 50 mM) with the final volume about 20 mL was incubated at 30 °C.

Next, the Δ^1^-dehydrogenations of hydrocortisone at substrate concentrations of 40–80 g/L were carried out with whole cells of *E. coli*/pET21a-PrKstD, and the data are shown in Figure 6. After 3 h, 40 and 50 g/L of hydrocortisone was converted to prednisolone with 95.5% and 93.7% conversion, respectively. Furthermore, at the concentrations of 60 g/L and 70 g/L, 92.5% of hydrocortisone was transformed to prednisolone after 3 h. Even about 90% conversion was reached at 80 g/L substrate concentration after 3 h, and the conversion increased slightly to 92.5% at 6 h. Longer duration of the process did not lead to a more complete conversion of the hydrocortisone. The remaining hydrocortisone was probably trapped in mixed crystals with prednisolone during the reaction, thereby decreasing its availability for reaction [39,40,41].

## 4. Discussion

**Figure 7:**
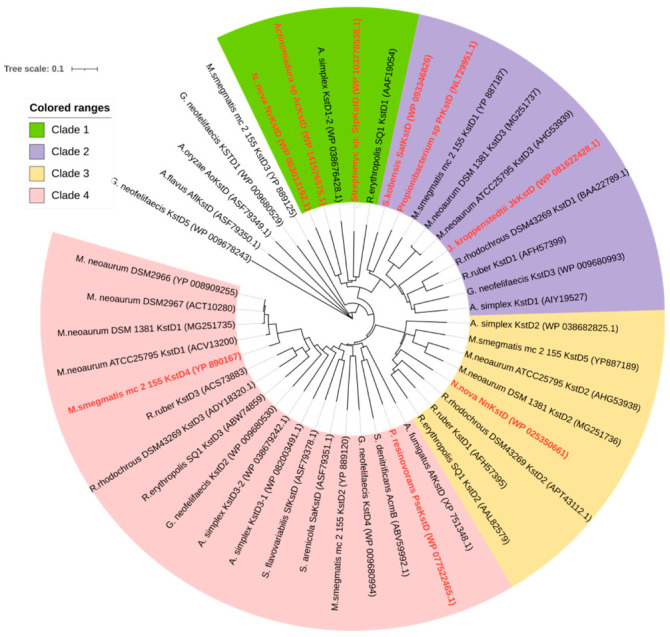
Phylogenetic tree of 3-ketosteroid dehydrogenases. The scale length was set at 0.1. Nine KstDs in this study is marked in red. Accession numbers from the GenBank database are shown in parentheses.

The properties of numerous KstDs from various origins were investigated in recent years [29,30,31,32]. These enzymes play important functions in steroid degradation and show significant application potential in the production of steroid drugs [5]. However, the application implementation is prevented by the limited availability of KstDs with high activity and wide substrate spectrum. The phylogenetic analysis (Figure 7) shows that KstD sequences may be clustered into at least four distinct groups. In order to expand the diversity of the KstDs toolbox with an aim of discovering new KstDs with high activity and wide substrate spectrum, in this study we selected nine KstDs that were scattered among these different groups. Alignment of amino acid sequences of these KstDs with SQKstD1 from *R. erythropolis* SQ1 shows that four residues (Y119, Y318, Y487, G491) essential for its activity in *R. erythropolis* SQ1 are conserved in these KstDs [33] (Figure S2). The consensus FAD-binding sequences with the characteristic GSGX_5–6_AX_2_AX_3_GLX_5_EX_5_GGXXAXSG are also evident [13]. These nine KstDs from various strains were tested toward substrates with diverse substituents on the steroidal nucleus, providing useful substrate profile information about these enzymes. These KstDs displayed activity toward steroids having a carbonyl group at C3 position. Among them, PrKstD displayed the highest specific activity and the broadest substrate profile toward the tested substrates.

The enzyme catalytic efficiency (*k*_cat_/*K*_m_) for AD was 24.9 × 10^6^ M^−1^ s^−1^, which was 19.2 times and 10.3 times higher than that of KsdD3 from *Arthrobacter simplex* [30] and AcmB from *Sterolibacterium denitrificans* [32]. The substrate profile of PrKstD was different to MsKstD1. For example, MsKstD1 preferred hydrocortisone and 9α-OH-AD rather than AD [29]. However, for PrKstD, the *k*_cat_/*K*_m_ for AD was much higher than those for hydrocortisone and 9α-OH-AD. Besides, an acetyl group at the C-17 position reduced the efficiency of the Δ^1^-dehydrogenation reaction catalyzed by PrKstD. More importantly, PrKstD is active toward the C6-methyl-substituted hydrocortisone and its kinetic parameters were determined. Although this is the first reported recombinant enzyme active toward 6α-methyl (17) and 6α-methyl-hydrocortisone (18), microbial dehydrogenation of these compounds has been previously reported [42,43,44] and the resulting products are effective and widely used medicaments for therapy of different rheumatic, allergic, cardiovascular and other diseases [45].

A wide substrate spectrum combined with the availability of the overexpression system, a cheap FAD regeneration system, high enzyme stability in the wide range of pH, and the possibility of applying whole cells as a biocatalyst make PrKstD very attractive for industrial application. The recombinant *E. coli* BL21 (DE3) harboring PrKstD enzyme showed impressive performance when used for Δ^1^-dehydrogenation of hydrocortisone. It displayed high activity at concentrations of hydrocortisone up to 80 g/L with the achievement of greater than 92.5% conversions. Compared to the Δ^1^-dehydrogenation of steroid substrates by heterologously expressing various KstDs reported in the literature (Table 4), it can be seen that the recombinant cells of pET21a(+)-PrKstD-BL21 reached the highest substrate concentration (80 g/L) and time-space productivity of 12.3 g/(L h). Therefore, PrKstD may serve as a promising biocatalyst for the effective transformation of hydrocortisone to prednisolone.

## 5. Conclusions

Enzymatic Δ^1^-dehydrogenation is known to be a key step in steroid metabolism and an important reaction in the synthesis of steroidal active pharmaceutical ingredients (APIs). The results from this study indicated that the recombinant KstDs of different origins showed preferences to different substrates. PrKstD was active toward the Δ^1^-dehydrogenation of a wide range of 3-ketosteroids with diverse substituents on the steroidal nucleus and C17 positon. In particular, PrKstD was demonstrated to catalyze the efficient transformation of hydrocortisone to prednisolone at up to 80 g/L substrate concentration, much higher than the previously reported results, advocating the possible utilization of the enzyme as a useful biocatalyst in large-scale production of prednisolone. How the PrKstD fine-tunes its substrate specificity is an intriguing subject for further investigation, which may be of interest for future biotechnological development and the production of specialty steroids.

## Figures and Tables

**Table 1:** Origins of KstDs.

Enzyme	Accession Number	Strain	Reference or Source
PrKstD	NLT29951.1	*Propionibacterium* sp.	This study ^a^
SatKstD	WP_093346826	*Saccharopolyspora kobensis*	This study
JkKstD	WP_081622428.1	*Jongsikchunia kroppenstedtii*	This study ^a^
StpKstD	WP_103778538.1	*Streptomyces* sp.	This study ^a^
NvKstD	WP_063013192.1	*Nocardia nova*	This study
AcKstD	WP_141576575.1	*Actinomadura* sp.	This study ^a^
PseKstD	WP_077522465.1	*Pseudomonas resinovorans*	This study ^a^
NnKstD	AHH19255	*Nocardia nova*	[27]
MsKstD4	YP_890167	*Mycobacterium smegmatis*	[27]

^a^ The gene was synthesized by General Biological Systems (Anhui) Co., Ltd.

**Table 2:** Substrate profiles of several KstD enzymes using cell-free extracts of recombinant *E. coli* cells.

Substrate	Specific Activity (U/mg)								
	PrKstD	SatKstD	JkKstD	StpKstD	NvKstD	AcKstD	PseKstD	NnKstD	MsKstD4
1	91.7	15.4	9.6	7.7	5.7	5.5	2.7	1.5	0.4
2	17.0	3.1	10.0	2.5	2.5	0.7	1.0	0.4	0.005
3	91.3	12.6	4.0	2.0	1.7	3.4	u.d.^a^	0.4	0.01
4	36.6	7.9	13.0	2.4	2.7	3.1	1.2	0.4	1.5
5	7.2	1.6	0.8	1.1	0.5	0.3	2.3	0	1.0
10	52.8	8.0	8.8	1.4	2.7	4.2	3.9	0.06	0.3
11	51.5	14.3	8.6	2.3	3.1	3.5	u.d.	0.1	0.03
12	43.9	10.6	9.8	1.6	2.3	2.8	1.5	0.01	0.2
13	41.1	10.1	8.7	0.7	3.5	1.8	0.9	0.01	0.07
14	30.3	16.3	16.9	1.3	2.7	4.1	u.d.	0.01	0.01
15	33.0	8.4	1.7	1.2	0.5	2.6	u.d.	u.d.	u.d.
17	0.6	0.06	u.d.	0.02	u.d.	u.d.	u.d.	u.d.	u.d.

u.d. the results were below detection limits. ^a^ The gene was synthesized by General Biological Systems (Anhui) Co., Ltd.

**Table 3:** Kinetic parameters of the purified PrKstD.

Substrate	*K*_m_ (µM)	*k*_cat_ (s^−1^)	*k*_cat_/*K*_m_ (×10^6^ M^−1^s^−1^)
1	9.9 ± 1.8	246.6 ± 6.2	24.9
2	21.5 ± 2.7	147.6 ± 3.7	6.9
3	28.6 ± 2.9	285.5 ± 6.5	10.0
4	9.2 ± 1.2	109.9 ± 1.9	11.9
5	36.6 ± 3.6	61.1 ± 1.5	1.7
6	137.7 ± 9.3	14.2 ± 0.3	0.1
7	8500.0 ± 1480	23.2 ± 1.9	0.003
8	95.0 ± 6.4	17.1 ± 0.3	0.2
9	155.0 ± 12	10.0 ± 0.2	0.06
10	31.9 ± 5.8	186.5 ± 11.5	5.8
11	21.9 ± 3.9	146.5 ± 5.0	6.7
12	29.0 ± 5.0	173.6 ± 6.0	6.0
13	20.8 ± 4.2	148.5 ± 6.3	7.1
14	23.7 ± 3.2	160.4 ± 4.6	2.4
15	164.5 ± 11.6	221.8 ± 4.2	1.3
16	211.7 ± 17.2	236.2 ± 5.8	1.1
17	343.3 ± 42.4	14.8 ± 0.6	0.04
18	443.3 ± 38.1	24.9 ± 0.7	0.06

**Table 4:** Recent works on Δ^1^-dehydrogenation of steroid substrates by heterologously expressing various KstDs.

Enzymes	Host Strains	Substrate, Conc. (g/L)	Time (h)	Biomass (g/L Wet Cells)	Productivity (g/(L h))	Reference
KsdD	*B. subtilis*	AD, 9	50	150	0.2	[25]
KsdD3 W299A	*E. coli* BL21	AD, 5	48	30	0.1	[30]
KstD2	*E. coli* BL21	AD, 30	8	50	3.7	[31]
MsKstD1	*E. coli* BL21	HC, 6	3	20	1.8	[29]
PrKstD	*E. coli* BL21	HC, 80	6	50	12.3	This study

## Data Availability

Not applicable.

## Supplementary Materials

The following are available online at https://www.mdpi.com/article/10.3390/microorganisms10030508/s1, Table S1: PCR primer sequences used in this study. Table S2: Organic solvent screening for the dehydrogenation of hydrocortisone catalyzed by *Escherichia. coli* cells overexpressing the PrKstD. Table S3: Purification of recombinant PrKstD. Table S4: Kinetic parameters of PrKstD for the oxidation of hydrocortisone with PMS/DCPIP. Figure S1: Protein purity of PrKstD by Ni^2+^ column. Figure S2: The sequence alignment of known KstD enzymes.
