# Supplementary material for: A New 3-Ketosteroid-Δ1–Dehydrogenase with High Activity and Broad Substrate Scope for Efficient Transformation of Hydrocortisone at High Substrate Concentration
Source: Microorganisms. 2022 Feb 25;10(3):508. doi: 10.3390/microorganisms10030508 (PMC8950399; doi:10.3390/microorganisms10030508)
Supplement: Supplementary file 1 [file microorganisms-10-00508-s001.zip › microorganisms-1570692-supplementary.pdf]

Supplementary Files

A new 3-ketosteroid-Δ^1–dehydrogenase with high activity and broad substrate scope for efficient transformation of hydrocortisone at high substrate concentration

Table S1 PCR primer sequences used in this study

Primer Name	Nucleotide sequence (5′-3′) [(a)]
SatkstD- forward	ATGGCGATCTGGGACGACGAGTG
SatkstD- reverse	TCAGCGGGTGAGCATGTCCAGC
NvkstD- forward	ATGACCTGGGATAATTCATACGACGTCATAGTGG
NvkstD - reverse	TCAGGATGCGGTGGCGTCCGC

(a) Primers were designed with NdeI and HindIII restriction sites to clone the *kstD* genes into the plasmids pET21a(+).

Table S2 Organic solvent screening for the dehydrogenation of hydrocortisone catalyzed by *Escherichia. coli* cells overexpressing the *PrkstD*

Organic solvent	Log P
Dimethyl sulfoxide	-1.30
1,4-dioxane	-1.10
Dimethyl formamide	-1.00
Methanol	-0.76
Ethanol	-0.24
Isopropyl alcohol	0.33
Tetrahydrofuran	0.49

Table S3 Purification of recombinant PrKstD

Purification step	Activity（U）	Specific activity（U/mg）	Purification (*n*-fold)	Yield (%)
Cell extract	18695	26	1.0	100
Ni-NTA	16342	188	7.2	87

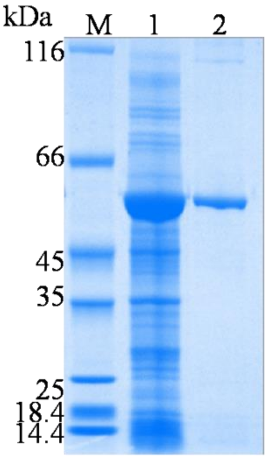

Figure S1 Protein purity of PrKstD by Ni^{2+} column. M, protein marker (kDa); 1, cell extract supernatant; 2, purified PrKstD from the elution step.

Table S4 Kinetic parameters of PrKstD for the oxidation of hydrocortisone with PMS/DCPIP

Electron acceptor	*K*m (μM)	k_{cat} (s^{-1})	k_{cat}/*K*m $s^{-1}μM^{-1}$	K_i(mM)
PMS/DCPIP	24.0±10	207.7±27	8.7	2.2±0.8

Enzyme activities were measured in a spectrophotometric assay at 600 nm in 50 mM potassium phosphate buffer pH 8.0, 0.5 mM hydrocortisone, 0.15 mM DCPIP, 0.02 μg PrKstD, 0-4 mM PMS.

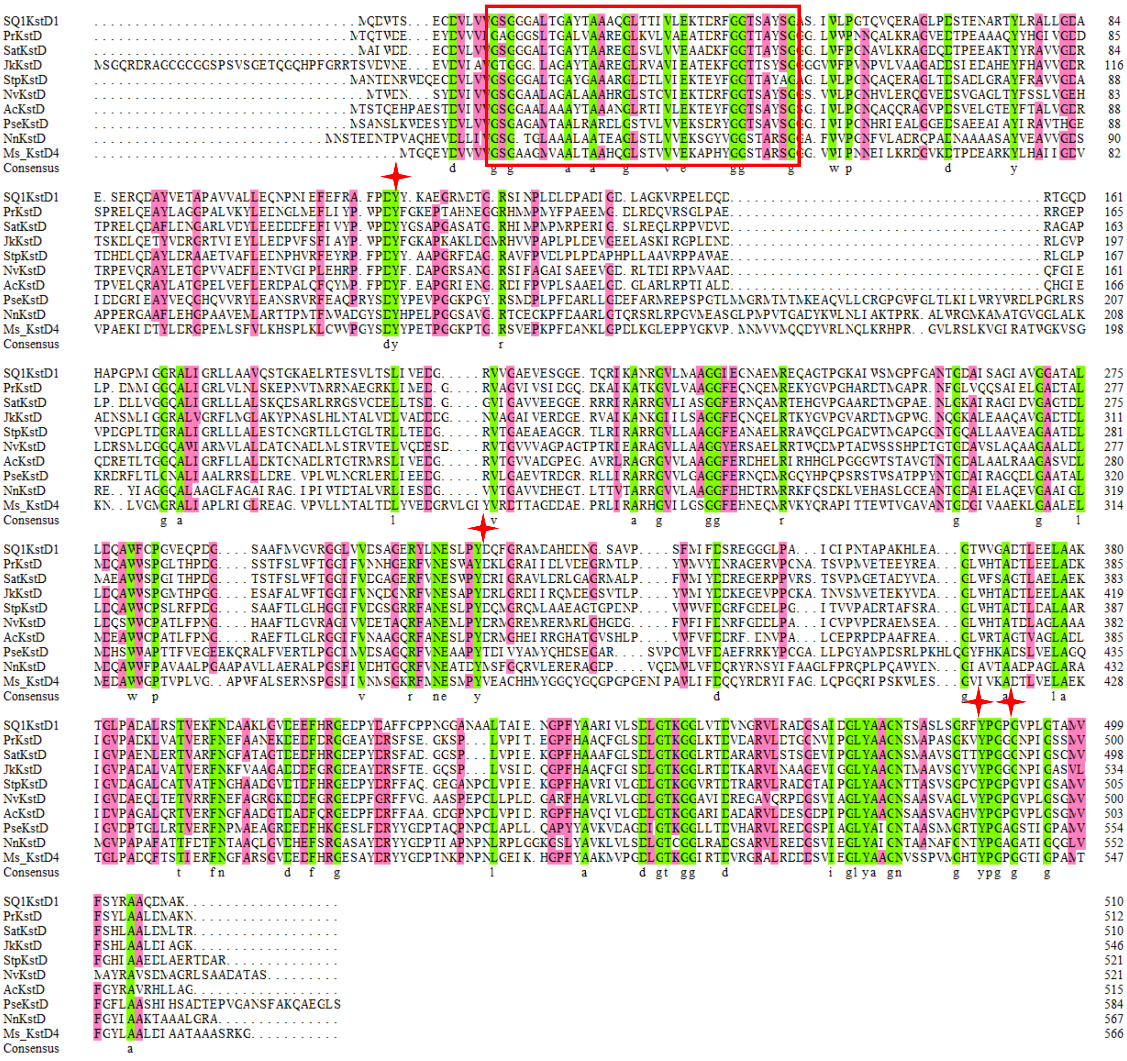

Figure S2 The sequence alignment of known KstD enzymes. SQ1 KstD from *Rhodococcus erythropolis* SQ1 (PDB entry 4C3Y). The FAD-binding domain is boxed in red. Active site residues essential for its activity in *R. erythropolis* SQ1 are indicated by red asterisks.
